# Investigating the Bond Strength of FRP Rebars in Concrete under High Temperature Using Gene-Expression Programming Model

**DOI:** 10.3390/polym14152992

**Published:** 2022-07-24

**Authors:** Muhammad Nasir Amin, Mudassir Iqbal, Fadi Althoey, Kaffayatullah Khan, Muhammad Iftikhar Faraz, Muhammad Ghulam Qadir, Anas Abdulalim Alabdullah, Ali Ajwad

**Affiliations:** 1 Department of Civil and Environmental Engineering, College of Engineering, King Faisal University, Al-Ahsa 31982, Saudi Arabia; kkhan@kfu.edu.sa (K.K.); 218038024@student.kfu.edu.sa (A.A.A.); 2 Department of Civil Engineering, University of Engineering and Technology Peshawar, Peshawar 25120, Pakistan; mudassiriqbal29@sjtu.edu.cn; 3 Department of Civil Engineering, Najran University, Najran 55461, Saudi Arabia; fmalthoey@nu.edu.sa; 4 Department of Mechanical Engineering, College of Engineering, King Faisal University, Al-Ahsa 31982, Saudi Arabia; mfaraz@kfu.edu.sa; 5 Department of Environmental Sciences, Abbottabad Campus, COMSATS University Islamabad, Abbottabad 22060, Pakistan; hashir785@gmail.com; 6 Civil Engineering Department, University of Management and Technology, Lahore 54770, Pakistan; ali.ajwad@umt.edu.pk

**Keywords:** GEP, FRP rebars, high temperature, AI, concrete

## Abstract

In recent times, the use of fibre-reinforced plastic (FRP) has increased in reinforcing concrete structures. The bond strength of FRP rebars is one of the most significant parameters for characterising the overall efficacy of the concrete structures reinforced with FRP. However, in cases of elevated temperature, the bond of FRP-reinforced concrete can deteriorate depending on a number of factors, including the type of FRP bars used, its diameter, surface form, anchorage length, concrete strength, and cover thickness. Hence, accurate quantification of FRP rebars in concrete is of paramount importance, especially at high temperatures. In this study, an artificial intelligence (AI)-based genetic-expression programming (GEP) method was used to predict the bond strength of FRP rebars in concrete at high temperatures. In order to predict the bond strength, we used failure mode temperature, fibre type, bar surface, bar diameter, anchorage length, compressive strength, and cover-to-diameter ratio as input parameters. The experimental dataset of 146 tests at various elevated temperatures were established for training and validating the model. A total of 70% of the data was used for training the model and remaining 30% was used for validation. Various statistical indices such as correlation coefficient (R), the mean absolute error (MAE), and the root-mean-square error (RMSE) were used to assess the predictive veracity of the GEP model. After the trials, the optimum hyperparameters were 150, 8, and 4 as number of chromosomes, head size and number of genes, respectively. Different genetic factors, such as the number of chromosomes, the size of the head, and the number of genes, were evaluated in eleven separate trials. The results as obtained from the rigorous statistical analysis and parametric study show that the developed GEP model is robust and can predict the bond strength of FRP rebars in concrete under high temperature with reasonable accuracy (i.e., R, RMSE and MAE 0.941, 2.087, and 1.620, and 0.935, 2.370, and 2.046, respectively, for training and validation). More importantly, based on the FRP properties, the model has been translated into traceable mathematical formulation for easy calculations.

## 1. Introduction

Due to its low cost, high strength, portability, and resistance to corrosion, insulation, and fatigue, fibre-reinforced plastic (FRP) provides unparalleled advantages in the construction of some types of infrastructure. The review of the current literature shows that the FRP bars manufactured by pultruding FRP fibres and combining them with vinylester and epoxy resin can be utilised as reinforcement materials for concrete constructions in hard climates and humid locations [1]. FRP bars can be made to replace carbon steel rebars as a reinforcement material for concrete structures. The most common exploitation of FRPs in concrete constructions include CFRP, BFRP, and GFRP. The mechanical characteristics, long-term endurance in adverse conditions, and creep/fatigue resistances of CFRP make it desirable; however, it is expensive. In contrast, GFRP and BFRP are less expensive because of easily available raw materials and advanced production techniques. However, they have weak mechanical characteristics and fatigue resistance. Additionally, glass fibre and basalt fibre degrade when exposed to the alkaline environment of concrete owing to hydrophobic interactions [2,3,4,5]. The bonding mechanism between the FRP rebars and the concrete matrix is noticeably distinct from that which occurs between steel rebars and concrete. As a consequence, a significant amount of time and effort has been invested in the investigation of this topic by means of both experimental and analytical research. There have been many different theories of interfacial bond-slip put forward, and the bond characteristic at ambient temperature has been adequately explained in many previous studies [6,7,8]. Despite this, FRP bars have a matrix of organic polymer materials, such as epoxy resin or vinylester resin, with a glass transition temperature of 60–130 degrees Celsius. Shear resistance of FRP rebars and their interlocking performance against concrete will significantly diminish due to the considerable weakening of the link between the matrix and the fibre [9]. Depending on the quantity of crosslinks and degree of crystallisation in FRP bars, the polymer loses its mechanical characteristics at high temperatures above the glass transition temperature (Tg). However, in FRP, the load is supported longitudinally by the inorganic fibres. The features in this direction are mostly determined by the characteristics of the fibres, which have superior thermal characteristics to the resin. For instance, Kumahara et al. [10] discovered that glass and carbon FRP rods’ tensile strength decreased by around 20% at a temperature of 250 °C, which was significantly higher than the resin’s Tg. Fresh concrete with improper placement and insufficient hydration includes free water, which evaporates above 100 °C and leads to fragmentation. Additionally, the bound water’s 300 °C evaporation in the hydrated components is accelerated by this circumstance [11]. An essential component of cement, calcium hydroxide, shrinks by 33 percent due to water loss and turns into quicklime at 530 °C. Concrete cracks as a consequence of the abrupt volume shift that occurs when the quicklime suddenly transforms from calcium hydroxide to calcium hydroxide as a result of the water being forced into the building during the fire [12]. At room temperature, the bond strength of concrete depends on surface of FRP rebar. Sand coating, wrapping with helical braid around the core, the quality of the polymer around the surface, and the formation of deformation may affect the bond strength between FRP and concrete. At a temperature equivalent to the glass-transition temperature of FRP, the polymer changes from a rigid condition to a soft and weaker state. Moreover, the interaction of water present in the concrete has already reached its boiling temperature at this temperature. Water from the concrete flows at the interface of concrete and polymer in the form of steam, leading to the degradation of bond strength owing to changes in concrete properties. Above the glass-transition temperature, loss of the bond strength is attributed to the mechanical degradation in resin material [13]. Furthermore, at elevated temperature, the bond of FRP-reinforced concrete can deteriorate depending on a number of factors, including the type of FRP bars used, its diameter, surface form, anchorage length, concrete strength, and cover thickness [14,15,16]. Previously, the researchers evaluated the behaviour of FRP under high temperature ranging between 20 °C and 375 °C [17], 23 °C to 600 °C [18], among others [19,20,21]. Some empirical formulas based on experimental studies have been proposed to express the variation of the bond characteristic with respect to temperature; however, it is necessary to improve the generality and accuracy of the existing computational models due to the limitations of the experimental conditions and the influence of multiple parameters.

In order to overcome the issues related with traditional theoretical modelling, the artificial intelligence (AI)-based modelling is gaining popularity in solving of many nonlinear complex engineering problems [22,23,24,25]. Several recent studies have calculated the strength of fibre-reinforced concrete using artificial neural networks (ANNs). Using 151 sets of pull-out experimental data of FRP-reinforced concrete at different temperatures, Huang et al. [26] built a backpropagating ANN network to predict the bond strength of FRP-reinforced concrete at high temperature. Congro et al. [27] utilised an ANN modelling technique to predict the flexural strength of fibre-reinforced concrete under bending-loading conditions. Lee and Lee [28] explored the ANN model to predict the shear strength of FRP flexural members without stirrups and discovered that the ANN model estimates the shear strength of FRP-reinforced concrete beams more precisely than existing formulae. Köroglu [29] investigated the bond strength of FRP-reinforced concrete using ANN by utilising the dataset of 406 beam specimens. The results depicted that the ANN showed superior predictive performance when compared with existing methods.

A few studies were also conducted to forecast the strength of FRP-reinforced retrofitted concrete beams using various AI techniques [30,31]. Nonetheless, the traditional AI methods such as ANN have a few shortcomings such as local minima trap issue [32,33,34,35]. In order to circumvent these issues, researchers utilised various other AI methods such as support vector machine (SVM), random forest (RF), gradient boosting machine (GBM), ensemble trees (ET), and extreme gradient boosting (EGB), etc., in predicting the strength of FRP-reinforced concrete [36,37,38,39]. Chen et al. [40] developed an ensemble learning-based gradient-boosted regression tree (GBRT) model to estimate FRP-concrete interfacial bond strength using 520 samples. The model’s performance was compared to empirical models and several others machine-learning algorithms. Through feature-importance analysis, this model’s rationale has been examined. The study’s model has the highest accuracy for forecasting bond strength in practice. Shahri and Mousavi [41] forecasted the bond strength of spliced GFRP bars by utilising several data-driven modelling techniques such as multivariate adaptive regression splines (MARS), M-5 trees, and Kriging. The results revealed that the MARS model has outperformed its counterparts. Concha [42] developed an ANN bond-strength prediction model for FRP-reinforced concrete using 184 hinged beam databases from previous trials. The suggested ANN model predicts FRP bond strength better than the prior studies and the available code equations. Although the accuracy of the models discussed above is reliable, their black box nature limits its practical implications.

In the recent past, gene-expression programing (GEP) has gained popularity in solving various engineering problems [43,44]. Murad et al. [45] predicted the flexural strength of FRP-reinforced concrete using GEP. One of the main advantages of GEP is the derivation of the closed form solution to the given problem [46]. Furthermore, GEP yields the output results in the form of a simple mathematical relationship, which maximises its significance in terms of future prediction easily. The resulting mathematical equation can be converted to any programming language for faster calculation of the results. Therefore, given the complexities of predicting the strength of FRP-reinforced concrete bars, this study utilises the GEP to predict the bond strength of FRP rebars at high temperature (mean ~151 degree Celsius). For this, 146 experimental datasets of FRP rebars in concrete at high temperature is established to train and test the developed GEP model. The model was evaluated using several statistical indices. The generalisation ability and robustness of the model were also evaluated using sensitivity analysis. More importantly, the developed model was also converted into a simple mathematical equation for the easy prediction of bond strength of FRP rebars in concrete at high temperature.

## 2. Materials and Methods

This section describes the approach used to calculate the bond strength of FRP rebars in concrete. A description of an experimental database is followed by a summary of the GEP model and its modelling technique. In addition, the criteria for evaluating the predictive strength of the model are also discussed in this section.

### 2.1. Experimental Database

For predicting the bond strength (*BS*), the input variables considered were temperature (*T*), failure mode (*FM*), fibre type (*FT*), bar surface (*B_s_*), bar diameter (*d_b_*), anchorage length (*L*), compressive strength ($f_{c}^{\prime }$), and cover-to-diameter ratio (*c/d_b_*) as input parameters. Mathematically,

(1) $$BS=f(T,\;FM,\;FT,\;B_{s},\;d_{b},\;L,\;f_{c}^{\prime },\;c/d_{b})$$

**Figure 1:**
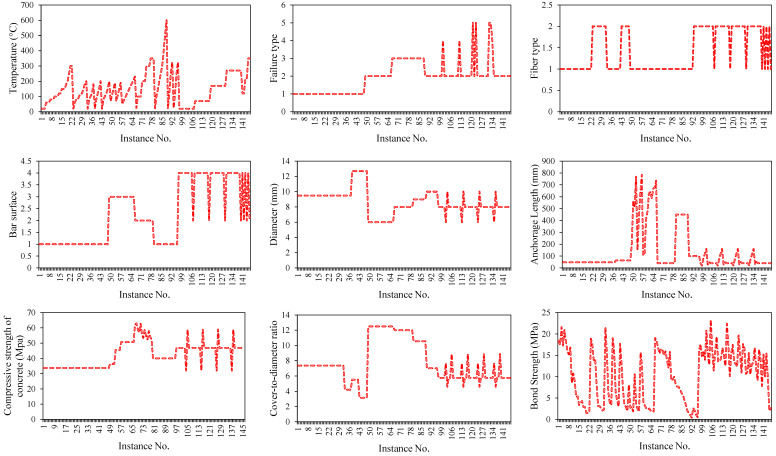
Input data used in the development of models.

The data were collected from the published literature [17,18,19,20,21], previously employed by Huang et al. [26] for developing ANN models. The descriptive statistics of all the parameters are summarised in Table 1. The contribution of these input factors in predicting the bond strength of FRP-reinforced concrete is clear from the previously established ACI formulae. For the sake of brevity, the mathematical formulae are not rehashed here but can easily be found in the literature [26]. Moreover, Figure 1 graphically represents the magnitude of all the variables. The standard deviation of the parameters revealed the utilisation of a broader range of variables in generating the experimental database. It may be noted that a few of the variables such as bar surface, failure mode, and bar type are coded as categorical variables in the GEP programming, whereas the rest of the parameters are modelled as continuous variables. For this, numerals 1 and 2 denote the GFRP and BFRP fibre types, respectively. The numbers 1–4 independently specify five surface variants of FRP bars, namely sand-coated (SC), ribbed (RB), fibre-wounded, and sand-coated (SC + SW), and sand-coated and ribbed (SC + RB). In addition, the debonding (D) between the FRP bars and the sand layer, the pull-out (P) of the FRP bars, the shear failure of the concrete (SF), the rupture of the FRP bars (R), and the splitting of the concrete (S) are considered and designated by the numbers 1–5, respectively. Table 2 summarises the codes applied for modelling the categorical variable in GEP modelling.

### 2.2. GEP Model Development

Ferreira [47] initially introduced GEP, whose forerunners include genetic algorithms (GA) and genetic programming (GP). It is a computer software contained in linear chromosomes of fixed length, and its knowledge-evaluation method is analogous to the biological one. Using the provided data, a mathematical function resembling a chromosome with several genes is constructed in this method. There are distinctions between GA, GP, and GEP, despite the fact that GEP performs symbolic regression using the majority of the genetic operators of GA and GP. Mathematical statements in GA are represented as symbolic strings of a constant length (chromosomes), but in GP they are represented as nonlinear entities of changing size and shape (parse trees). However, in GEP, they are expressed as expression trees (ET) of varying sizes and forms, with the strings themselves being encoded as plain strings of constant length [48].

**Figure 2:**
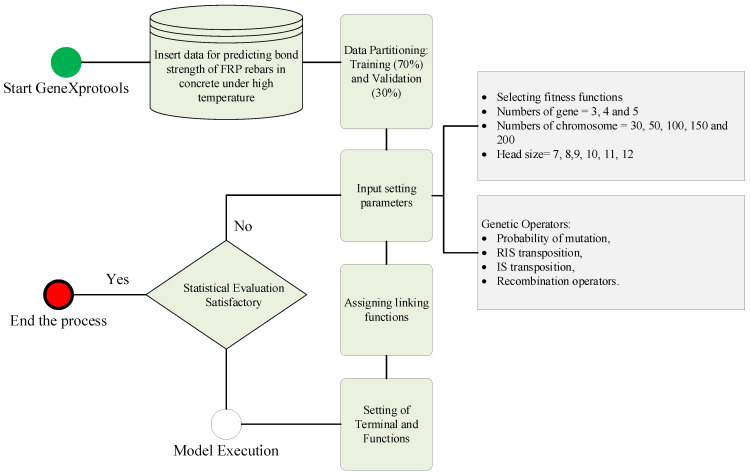
GEP framework for predicting the FRP bond strength.

For modelling the BS of the FRP-reinforced concrete, the shape and size of ETs were adjusted according to the learning of the GEP model. The 70:30 data split was used to train and test the GEP model. It may be noted that the training dataset is used to train the model and testing dataset to validate the model. Following that, the configuration parameters were tweaked to produce a high-performance model. The root-mean-square error (RMSE) was selected as a fitness function, and the number of genes, chromosomes, and head size were altered, systemically. Furthermore, genetic operators such as selection, mutation probability, transposition (IS and RIS), and cross-over (recombination) operators were established in accordance with previous literature [49,50]. The +, −, sqrt, and x^2^ functions were used to establish relationships within ETs, while the + function was used to establish relationships between ETs. The model was trained until the optimal solution is established. In other words, it means that the best fit is the moment at which the model’s performance in terms of correlations and error indices stops improving. It may be noted that the performance of the validation data was also checked to prevent model over-fitting. Once the optimal performance was achieved, the model’s ability to produce mathematical equations was discontinued. Figure 2 represents the framework of GEP proposed for this study.

### 2.3. Evaluation Criteria

Four statistical indices, namely root-mean-square error (RMSE), mean absolute error (MAE), coefficient of determination (R^2^), and Pearson’s coefficient^®^, were utilised to appraise the accuracy of the developed GEP model. All these parameters are well-recognised indicators for evaluating the strength of data-driven models [51,52,53,54,55].

## 3. Results and Discussion

The results of this study are presented in this Section. After a detailed explanation of how changing genetic variables affects a model’s output, the model’s actual results are presented in Section 3.1, Section 3.2 and Section 3.3. Finally, the results of a parametric study are presented in order to evaluate the relative influence of contributing variables on the bond strength of FRP rebars in high-temperature concrete.

### 3.1. Effect of Genetic Variables

**Figure 3:**
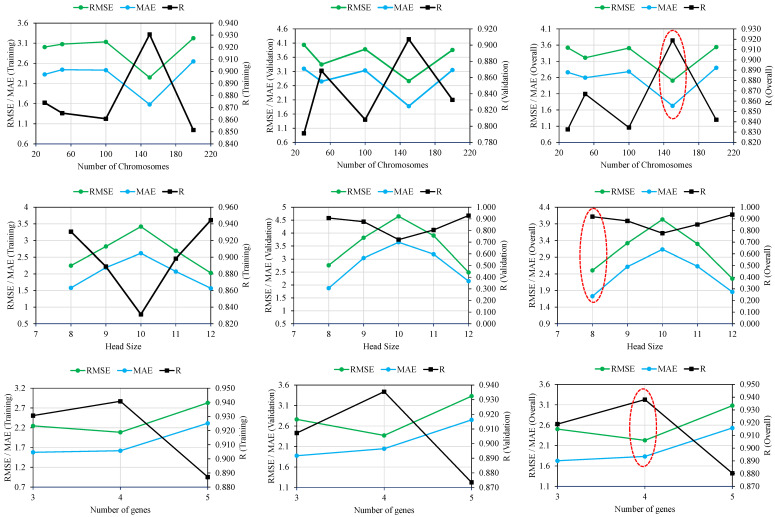
Effect of genetic variables on the performance of the undertaken trials.

**Figure 4:**
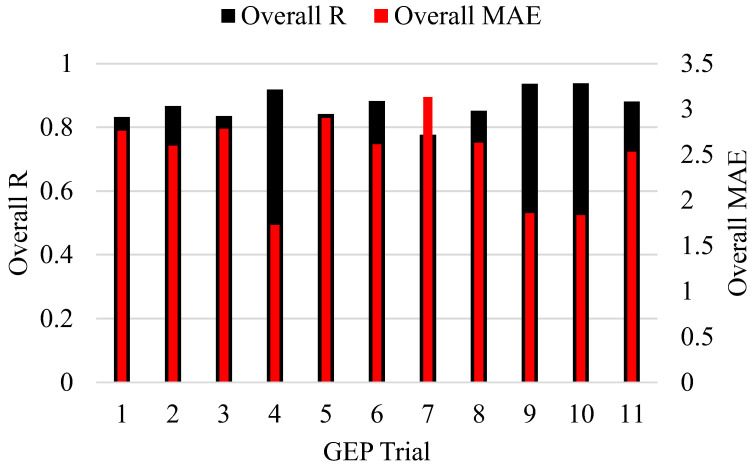
Comparison of the developed trials.

Adjustments to genetic factors and their associated changes in R, MAE, and RMSE are shown in Figure 3. The best model was selected by averaging the training and validation data’s R, MAE, and RMSE values, despite the fact that model performance varied over the two phases. The initial experiment involved changing the number of chromosomes by 30, 50, 100, 150, and 200, while keeping the number of genes and the size of the head at 3 and 8, respectively. Table 2 shows that changing the number of chromosomes from 30 to 50 decreased the R values for the training data from 0.764 to 0.749 and increased them for the validation data from 0.626 to 0.754. Both MAE and RMSE values followed a consistent pattern as the number of chromosomes increased from 30 to 50. Model performance was not enhanced by increasing the number of chromosomes from 50 to 100. The models with four different chromosomal counts performed best at a magnitude of 150. Head sizes were increased from 8 to 9, 10, 11, and 12 in successive experiments, while the chromosome count was maintained at 150. There was a clear upward trend in performance for the models as the head size increased from 8 to 9, and then back down to 10. To be specific, a head size of 8 produced the best outcomes. Therefore, in order to fine-tune the ensuing experiments with varied numbers of genes, two parameters—chromosome number and head size—were optimised at 150 and 8, respectively. It was found that adding more genes reduces model performance, and that adding more than five genes introduces additional complications to the output equation [44]. According to Figure 3, optimal model performance was achieved with four genes. The optimal values of hyperparameters for the number of chromosomes, head size, and genes in this study are 150, 8, and 4, respectively. It was shown that the trial and access techniques determine the GEP modelling settings; thus, these must be discovered by extensive experimentation by adjusting the genetic parameters [24]. Figure 4 demonstrates that the best trial observed was trial number 10, with magnitudes of overall R, MAE of 0.940 and 1.71, respectively.

### 3.2. Performance of the Undertaken Trials

The developed GEP model was evaluated on the basis of statistical indices described in Section 2.3. Thereafter, the results were analysed using regression plots and error analysis of results, and predicted vs. experimental ratio. It is noteworthy that this analysis was undertaken using the results of the optimum GEP model (Trial 10).

#### 3.2.1. Statistical Indices Analysis

The results of all the statistical parameters (R, R^2^, MAE, and RMSE) for all the trials are summarised in Table 3. For the training data and validation data, the minimum value of R was found to be 0.691 and 0.521 for trial 7, respectively. For the training data of trial 7, the maximum value of MAE was found to be 2.617, while for the validation data, it was found to be 3.647. Moreover, the values of RMSE for both the datasets were 3.417 and 4.647, respectively. The best values, that is, 0.941, 0.885, 1.620, and 2.087, respectively, for R, R^2^, MAE, and RMSE were obtained for trial 10. The statistical analysis of all the trials revealed a close correlation between experimental and projected results; nevertheless, the performance of trial 10 findings outperformed its counterparts. Therefore, the model from trial 10 is more reliable for future prediction of bond strength of FRP rebars in concrete at high temperatures. 

#### 3.2.2. Regression Plot Analysis and Error Analysis

**Figure 5:**
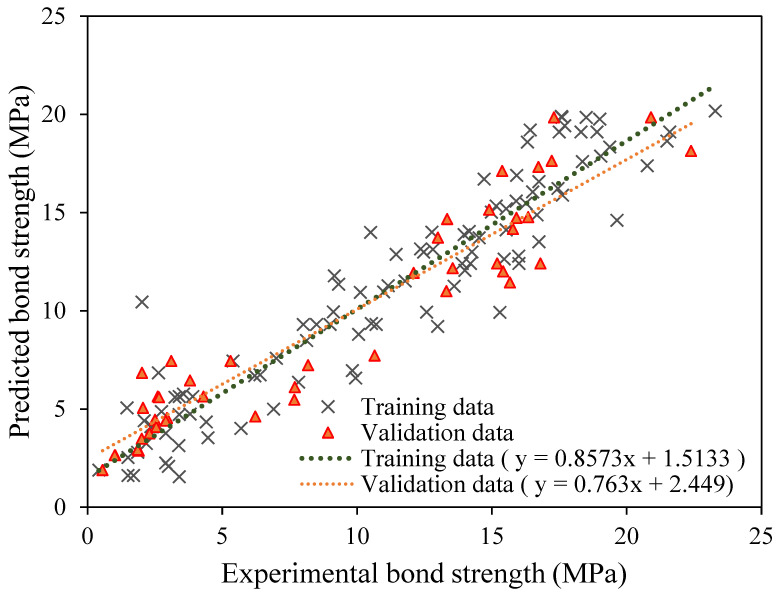
Comparison of the regression slopes for the training and validation data of the optimised trial No. 10.

**Figure 6:**
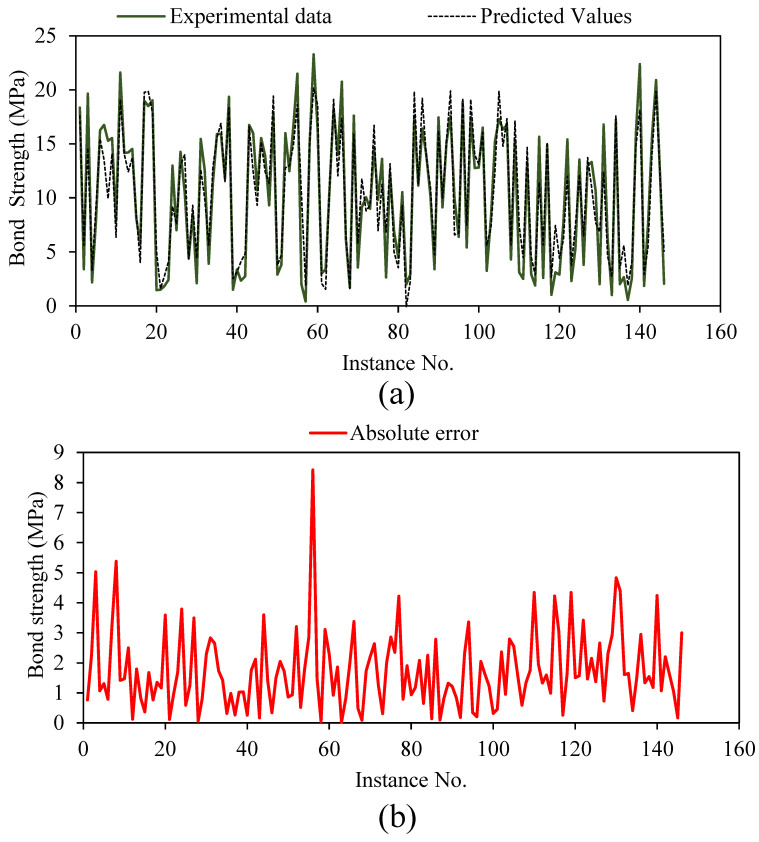
Error analysis of the optimised trial. (**a**) Tracing of experimental results by the predictions; (**b**) absolute errors for trial No. 10 predictions.

The analysis of the regression plot is shown in Figure 5, where experimental data are plotted along the abscissa (*x*-axis), and projected bond-strength values are plotted along the ordinate (*y*-axis). Exact mapping at a 1:1 ratio is the best-case scenario for these sorts of charts. Amin et al. [2] investigate the GEP’s potential for predicting the interfacial bond strength of externally bonded FRP laminates on grooves with concrete prisms and find the slopes of the regression line to be 0.99 and 0.96, respectively, for the training and validation datasets. When comparing the actual and expected values, a value of 0.8 or higher indicates a highly substantial correlation. The slope of the regression line in this study is 0.867 in the training dataset and 0.763 in the validation dataset. The slope is near to 0.8, indicating a satisfactory match between observed and projected values, even though it is slightly lower in the validation dataset. Figure 6 displays the error analysis plot. The greatest amount of variation in absolute value is shown in the figure to fall between 0 and 4.0 MPa. The errors are well-distributed along the *x*-axis, showing that model is neither over- nor undertrained (Figure 6).

#### 3.2.3. Predicted vs. Experimental Ratio

**Figure 7:**
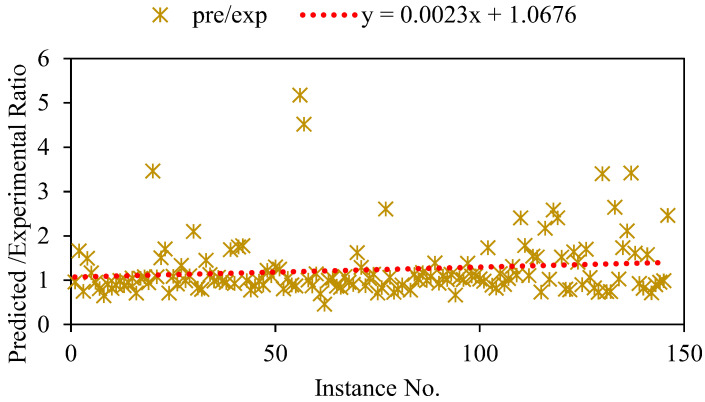
Predicted/experimental ratio for the optimum trial.

According to the results shown in Section 3.2, the trial 10 model was the most accurate of the models generated in this investigation. Therefore, in order to more clearly illustrate the accuracy, the data of trial 10 are presented as predicted/experimental values. Our study shows that most observations fall within the range of 0.7 to 1.3, and that 90% are more likely to be slightly underforecasted than overanticipated (Figure 7). The predicted/experimental results supplemented the statistical evaluation which showed that the developed GEP model predicted the bond strength with reasonable accuracy.

### 3.3. GEP Formulations

**Figure 8:**
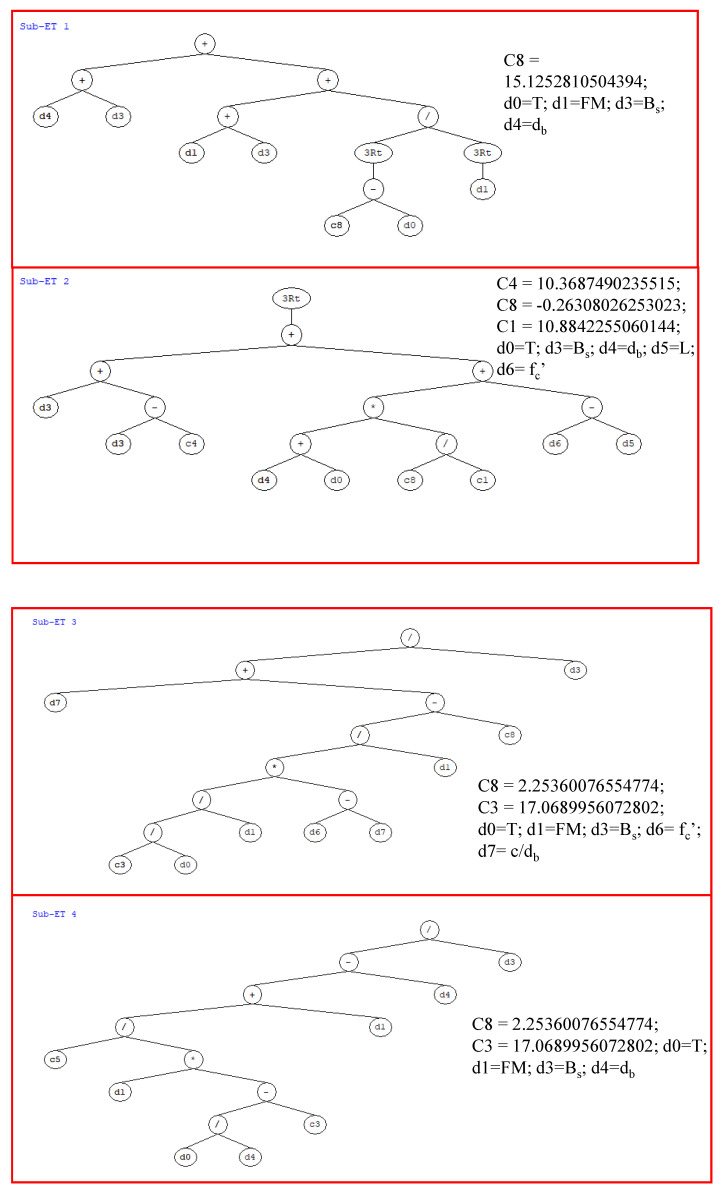
Expression tree obtained from optimised trial in GEP modelling.

The model derived from the GEP study was used to derive simple mathematical equations for predicting the bond strength of FRP rebars in concrete under high temperature. Figure 8 depicts the ETs retrieved from the GEP model, which was utilised to generate prediction equations (Equations (3)–(6)). Since four genes were tested in Trial 10, each ET is made up of four sub-ETs. Each subconstant, ETs denoted by the numbers C1, C2, C3, etc., are shown in Figure 7. Utilizing these constants and linking functions (+, −, ×, /) in each sub-ET, the mathematical equation was formulated. Each input parameter (*d_b_* is the diameter of bar; *B_s_* is the bar surface; *FM* is failure mode; *T* is the temperature; $f_{c}^{\prime }$ is the concrete compressive strength; *L* is the anchorage length; and $\frac{c}{d_{b}}$ is the cover-to-diameter ratio) is identified in Equation (2) as being used to predict the value of bond strength.

(2) Y=a+b+c+d

(3) $$a=\left(\left(d_{b}+B_{s}\right)+\left(\left(FM+B_{s}\right)+\frac{\sqrt[{3}]{\left(15.125-T\right)}}{\sqrt[{3}]{FM}}\right)\right)$$

(4) $$b=\sqrt[{3}]{(\left(B_{s}+\left(B_{s}-10.37\right)\right)+\left(\left(\left(d_{b}+T\right)\times \frac{-0.26}{10.884}+\left(f_{c}^{\prime }-L\right)\right)\right)}$$

(5) $$c=\frac{\left(c/d_{b}+\left(\frac{(\left(\frac{17.067/T}{FM}\right)\times (f_{c}^{\prime }-{\frac{c}{d}}_{b}))}{FM}\right)-2.25\right)}{B_{s}}$$

(6) $$d=\frac{\frac{18.374}{\left(FM\times \left(\frac{T}{d_{b}}-4.42\right)+FM\right)}-d_{b}}{B_{s}}$$

### 3.4. Parametric Analysis

**Figure 9:**
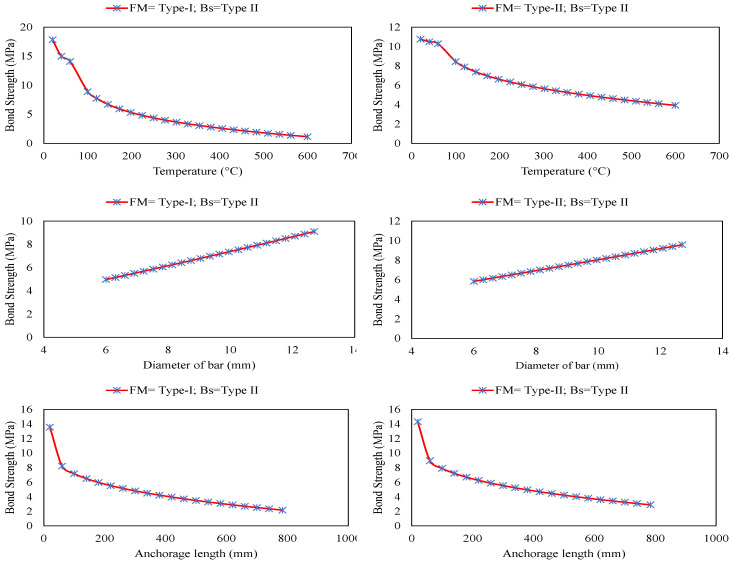

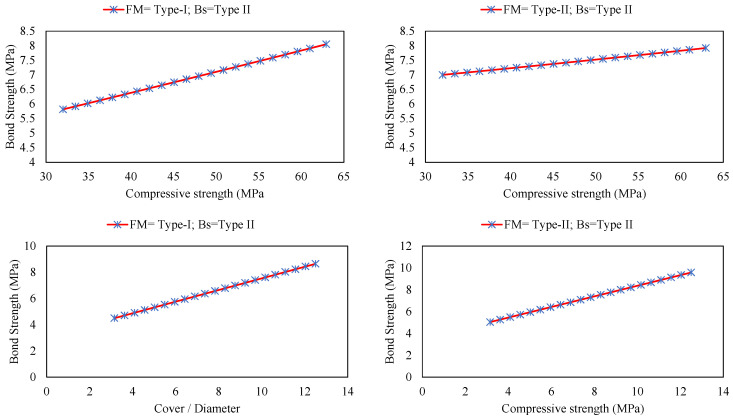
Parametric study of the GEP model showing variation of bond strength with change in input variable for Type I (debonding) and Type II (pull-out) failure modes corresponding to Type-II bar surface (Ribbed).

Scholars have suggested that the accuracy and dependability of AI-based models should not be evaluated based only on statistical criteria [56,57,58,59,60]. A solid and trustworthy model provides a good fit to the calibration data and produces predictions that rationally account for the underlying physical behaviour of the researched system. In light of this criterion, the generalisation ability of the constructed GEP model has been carefully evaluated in this work using parametric analysis. Figure 9 shows the parametric study of the GEP model showing variation of bond strength with change in input variable for Type I (debonding) and Type II (pull-out) failure modes corresponding to Type-II bar surface (Ribbed). For this, each input variable is varied between the minimum and maximum value (Table 2) by keeping the rest of the variables at its mean value. A similar method has been utilised by the previous researchers to assess the generalisation ability of ML models [59,61,62,63]. As can be observed from the figure that for both the failure mode (debonding and pull-out) increase in temperature and anchorage length cause the decrease in the bond strength. Previous experimental studies have also depicted similar trends, whereas an increase in the diameter of bar, compressive strength, and cover-to-diameter ratio cause the increase in the bond strength of the FRP rebar in concrete. Although these trends conform the underlying behaviour of investigated system, for the optimal magnitude of these factors, a comprehensive investigation based on numerous experiments is required.

## 4. Conclusions

Using the GEP approach, this study predicted the bond strength of FRP bars and concrete at elevated temperatures. The dataset was established by evaluating the bond strength of FRP rebars in high-temperature concrete by performing 146 experimental tests. Thereafter, the GEP modelling technique has been utilised to train and test the data. Based on the comparison of measured and predicted results, the following general inferences can be made:

1. The proposed GEP model has the potential to be used as a tool for extracting features and making predictions in very intricate nonlinear engineering systems. The GEP architecture needs to be optimised via hit-and-trial method based on the dataset’s size and complexity. Dependence on the amount and quality of the dataset is a key factor in the GEP model’s utility and accuracy.
2. Measures of statistical performance including R, RMSE, and MAE were applied to both the training data and the validation data to assess the quality of the models. R = 0.941, MAE = 1.620, and RMSE = 2.087 for training, and 0.935, 2.046, and 2.370 for validation, according to the statistical indices, demonstrates that the established GEP model can accurately estimate bond strength.
3. The parametric analysis shows that the developed GEP model can accurately predict the response of different input variables on the strength of FRP bars. Due to the limited data, this analysis is limited to ribbed bars.

More importantly, the mathematical equations were developed that can be used by the researchers to predict the bond strength of FRP rebars in concrete at high temperature.

## Figures and Tables

**Table 1:** Descriptive statistics of input data used in GEP modelling.

Variable	Input Variables								Target
	Temperature	Failure Mode	Fibre Type	Bar Surface	Diameter	Anchorage Length	Compressive Strength	Cover-to-Diameter Ratio	Bond Strength
Identification	*T*	*FM*	*FT*	*B_s_*	*d_b_*	*L*	$f_{c}^{\prime }$	*c/d_b_*	*BS*
Descriptive statistics	°C	-	-	-	mm	mm	MPa	-	MPa
Mean	150.99	1.95	1.43	2.27	8.66	135.61	42.52	7.80	10.32
Standard Error	8.74	0.07	0.04	0.11	0.14	15.46	0.69	0.23	0.53
Median	125.00	2.00	1.00	2.00	8.00	47.50	42.76	7.37	10.84
Mode	20.00	2.00	1.00	1.00	8.00	40.00	33.70	5.75	3.40
Standard Deviation	105.59	0.90	0.50	1.30	1.68	186.75	8.30	2.79	6.35
Sample Variance	11,149.45	0.81	0.25	1.69	2.81	34,875.73	68.89	7.81	40.34
Kurtosis	1.70	2.05	−1.95	−1.65	0.66	3.04	−0.75	−0.91	−1.36
Skewness	1.06	1.20	0.28	0.31	0.52	2.07	0.45	0.54	−0.01
Range	580.00	4.00	1.00	3.00	6.70	764.00	30.93	9.35	22.87
Minimum	20.00	1.00	1.00	1.00	6.00	20.00	32.00	3.15	0.42
Maximum	600.00	5.00	2.00	4.00	12.70	784.00	62.93	12.50	23.29
Count	146.00	146.00	146.00	146.00	146.00	146.00	146.00	146.00	146.00
Confidence Level (95.0%)	17.27	0.15	0.08	0.21	0.27	30.55	1.36	0.46	1.04

**Table 2:** Code applied for modelling categorical variables in GEP models.

Categorical Input Variable	Property	Code
Bar Surface (Bs)	Sand-coated (SC)	1
	Ribbed (RB)	2
	Fibre-wounded (SW)	
	SC + SW	3
	SC + RB	4
Failure mode (FM)	Debonding (D)	1
	Pull-out (P)	2
	Shear failure of concrete (SF)	3
	FRP rupture (R)	4
	Splitting of concrete (S)	5
Type of FRP	GFRP	1
	BFRP	2

**Table 3:** Statistical evaluation of the trials undertaken in finding optimised model.

Trial No.	Used Variables	No. of Chromosomes	Head Size	Number of Genes	Constants per Gene	No. of Literals	Program Size	Training Dataset					Validation Dataset				
								Best Fitness	RMSE	MAE	R^2^	R	Best Fitness	RMSE	MAE	R^2^	R
1	8	30	8	3	10	17	41	249.7	3.004	2.326	0.764	0.874	198.550	4.036	3.197	0.626	0.791
2	8	50	8	3	10	15	42	245.3	3.076	2.442	0.749	0.865	229.980	3.348	2.750	0.754	0.868
3	8	100	8	3	10	15	35	241.9	3.133	2.430	0.741	0.861	204.770	3.883	3.136	0.653	0.808
4	7	150	8	3	10	13	41	307.9	2.248	1.580	0.866	0.931	265.820	2.762	1.876	0.823	0.907
5	8	200	8	3	10	16	37	236.8	3.223	2.649	0.725	0.851	205.790	3.859	3.152	0.693	0.832
6	8	150	9	3	10	20	47	261.5	2.820	2.188	0.790	0.889	207.110	3.820	3.040	0.767	0.876
7	8	150	10	3	10	19	45	226.4	3.417	2.617	0.691	0.831	177.080	4.647	3.647	0.521	0.722
8	8	150	11	3	10	16	45	270.8	2.692	2.071	0.807	0.898	203.860	3.905	3.186	0.647	0.804
9	8	150	12	3	10	16	55	330.2	2.028	1.567	0.892	0.944	286.760	2.487	2.148	0.862	0.928
10	7	150	8	4	10	25	65	323.9	2.087	1.620	0.885	0.941	296.750	2.370	2.046	0.875	0.935
11	7	150	8	5	10	20	83	261.0	2.830	2.314	0.787	0.887	230.950	3.329	2.750	0.763	0.873

## Data Availability

The data used in this research have been properly cited and reported in the main text.

## References

[1] Sun L., Yang Z., Zhu C., Zhang C., Hui X. (2020). Study on Bonding Properties of Reinforced Composite Concrete Structure with Fiber Materials. Tumu Gongcheng Xuebao/China Civ. Eng. J..

[2] Amin M.N., Iqbal M., Khan K., Qadir M.G., Shalabi F.I., Jamal A. (2022). Ensemble Tree-Based Approach towards Flexural Strength Prediction of FRP Reinforced Concrete Beams. Polymers.

[3] Li C., Yin X., Liu Y., Guo R., Xian G. (2020). Long-Term Service Evaluation of a Pultruded Carbon/Glass Hybrid Rod Exposed to Elevated Temperature, Hydraulic Pressure and Fatigue Load Coupling. Int. J. Fatigue.

[4] Wang Z., Zhao X.L., Xian G., Wu G., Raman R.K.S., Al-Saadi S. (2018). Effect of Sustained Load and Seawater and Sea Sand Concrete Environment on Durability of Basalt- and Glass-Fibre Reinforced Polymer (B/GFRP) Bars. Corros. Sci..

[5] Li C., Guo R., Xian G., Li H. (2020). Innovative Compound-Type Anchorage System for a Large-Diameter Pultruded Carbon/Glass Hybrid Rod for Bridge Cable. Mater. Struct. Constr..

[6] Taerwe L. (2020). Analytical Modelling of Bond between FRP Reinforcing Bars and Concrete. Non-Metallic Reinforcement for Concrete Structures.

[7] Tighiouart B., Benmokrane B., Gao D. (1998). Investigation of Bond in Concrete Member with Fibre Reinforced Polymer (FRP) Bars. Constr. Build. Mater..

[8] Malvar L.J. (1995). Tensile and Bond Properties of GFRP Reinforcing Bars. ACI Mater. J..

[9] Guo R., Xian G., Li F., Li C., Hong B. (2022). Hygrothermal Resistance of Pultruded Carbon, Glass and Carbon/Glass Hybrid Fiber Reinforced Epoxy Composites. Constr. Build. Mater..

[10] Kumahara S., Masuda Y., Tanano H., Shimizu A. (1993). Tensile strength of continuous fiber bar under high temperature. Special Publication. ACI Symp. Pap..

[11] Ahmet B., Rüstem G.Ü.L. (2009). Donatı-beton aderansı, yüksek sıcaklıkların beton dayanımına ve aderansa etkileri konusunda bir derleme. Tübav Bilim Dergisi.

[12] Khoury G.A. (1996). Performance of Heated Concrete-Mechanical Properties.

[13] Katz A., Berman N. (2000). Modeling the effect of high temperature on the bond of FRP reinforcing bars to concrete. Cem. Concr. Compos..

[14] Spagnuolo S., Meda A., Rinaldi Z., Nanni A. (2018). Residual Behaviour of Glass FRP Bars Subjected to High Temperatures. Compos. Struct..

[15] Naser M.Z., Uppala V.A. (2020). Properties and Material Models for Construction Materials Post Exposure to Elevated Temperatures. Mech. Mater..

[16] Robert M., Benmokrane B. (2010). Behavior of GFRP Reinforcing Bars Subjected to Extreme Temperatures. J. Compos. Constr..

[17] Hamad R.J.A., Megat Johari M.A., Haddad R.H. (2017). Mechanical properties and bond characteristics of different fiber reinforced polymer rebars at elevated temperatures. Constr. Build. Mater..

[18] Özkal F.M., Polat M., Yağan M., Öztürk M.O. (2018). Mechanical properties and bond strength degradation of GFRP and steel rebars at elevated temperatures. Constr. Build. Mater..

[19] El-Gamal S. (2014). Bond strength of glass fiber-reinforced polymer bars in concrete after exposure to elevated temperatures. J Reinf. Plast. Compos..

[20] Wang Y.L. (2013). Experimental Study on Tensile Property of FRP Bars and Bond Behavior between FRP Bars and Concrete after High Temperature.

[21] Lu X.L., Zhou C.D., Jin Y. (2007). Test study on bond behavior between GFRP bar andconcrete in high temperature. J. Build. Struct..

[22] Jalal F.E., Xu Y., Iqbal M., Jamhiri B., Javed M.F. (2021). Predicting the Compaction Characteristics of Expansive Soils Using Two Genetic Programming-Based Algorithms. Transp. Geotech..

[23] Trong D.K., Pham B.T., Jalal F.E., Iqbal M., Roussis P.C., Mamou A., Ferentinou M., Vu D.Q., Dam N.D., Tran Q.A. (2021). On Random Subspace Optimization-Based Hybrid Computing Models Predicting the California Bearing Ratio of Soils. Materials.

[24] Khan K., Jalal F.E., Iqbal M., Khan M.I., Amin M.N., Al-Faiad M.A. (2022). Predictive Modeling of Compression Strength of Waste PET/SCM Blended Cementitious Grout Using Gene Expression Programming. Materials.

[25] Bardhan A., Samui P., Ghosh K., Gandomi A.H., Bhattacharyya S. (2021). ELM-Based Adaptive Neuro Swarm Intelligence Techniques for Predicting the California Bearing Ratio of Soils in Soaked Conditions. Appl. Soft Comput..

[26] Huang L., Chen J., Tan X. (2022). BP-ANN based bond strength prediction for FRP reinforced concrete at high temperature. Eng. Struct..

[27] Tran T.H., Dam N.D., Jalal F.E., Al-Ansari N., Ho L.S., Van Phong T., Iqbal M., Van Le H., Nguyen H.B.T., Prakash I. (2021). GIS-Based Soft Computing Models for Landslide Susceptibility Mapping: A Case Study of Pithoragarh District, Uttarakhand State, India. Math. Probl. Eng..

[28] Lee S., Lee C. (2014). Prediction of shear strength of FRP-reinforced concrete flexural members without stirrups using artificial neural networks. Eng. Struct..

[29] Congro M., Monteiro V.M.D.A., Brandão A.L.T., dos Santos B.F., Roehl D., Silva F.D.A. (2021). Prediction of the Residual Flexural Strength of Fiber Reinforced Concrete Using Artificial Neural Networks. Constr. Build. Mater..

[30] Alam M.S., Sultana N., Hossain S.Z. (2021). Bayesian optimization algorithm based support vector regression analysis for estimation of shear capacity of FRP reinforced concrete members. Appl. Soft Comput..

[31] Köroglu M.A. (2019). Artificial Neural Network for Predicting the Flexural Bond Strength of FRP Bars in Concrete. Sci. Eng. Compos. Mater..

[32] Lee S., Moy S. (2007). A Method for Predicting the Flexural Strength of RC Beams Strengthened with Carbon Fiber Reinforced Polymer. J. Reinf. Plast. Compos..

[33] Pham H., Al-Mahaidi R. (2004). Assessment of Available Prediction Models for the Strength of FRP Retrofitted RC Beams. Compos. Struct..

[34] Raja M.N.A., Shukla S.K. (2021). Multivariate Adaptive Regression Splines Model for Reinforced Soil Foundations. Geosynth. Int..

[35] Raja M.N.A., Shukla S.K. (2020). An Extreme Learning Machine Model for Geosynthetic-Reinforced Sandy Soil Foundations. Proc. Inst. Civ. Eng.-Geotech. Eng..

[36] Kardani N., Bardhan A., Gupta S., Samui P., Nazem M., Zhang Y., Zhou A. (2021). Predicting Permeability of Tight Carbonates Using a Hybrid Machine Learning Approach of Modified Equilibrium Optimizer and Extreme Learning Machine. Acta Geotech..

[37] Kardani N., Bardhan A., Samui P., Nazem M., Zhou A., Armaghani D.J. (2021). A Novel Technique Based on the Improved Firefly Algorithm Coupled with Extreme Learning Machine (ELM-IFF) for Predicting the Thermal Conductivity of Soil. Eng. Comput..

[38] Cakiroglu C., Islam K., Bekdaş G., Kim S., Geem Z.W. (2022). Interpretable Machine Learning Algorithms to Predict the Axial Capacity of FRP-Reinforced Concrete Columns. Materials.

[39] Kaveh A., Javadi S.M., Moghanni R.M. (2022). Shear Strength Prediction of FRP-Reinforced Concrete Beams Using an Extreme Gradient Boosting Framework. Period. Polytech. Civ. Eng..

[40] Abbasloo A.A., Shayanfar M.A., Pahlavan H., Barkhordari M.A., Hamze-Ziabari S.M. (2019). Prediction of Shear Strength of FRP-Reinforced Concrete Members Using a Rule-Based Method. Mag. Concr. Res..

[41] Deifalla A., Salem N.M. (2022). A Machine Learning Model for Torsion Strength of Externally Bonded FRP-Reinforced Concrete Beams. Polymers.

[42] Chen S.Z., Zhang S.Y., Han W.S., Wu G. (2021). Ensemble Learning Based Approach for FRP-Concrete Bond Strength Prediction. Constr. Build. Mater..

[43] Shahri S.F., Mousavi S.R. (2021). Bond Strength Prediction of Spliced GFRP Bars in Concrete Beams Using Soft Computing Methods. Comput. Concr..

[44] Concha N.C. (2022). Neural Network Model for Bond Strength of FRP Bars in Concrete. Structures.

[45] Jalal F.E., Xu Y., Iqbal M., Javed M.F., Jamhiri B. (2021). Predictive Modeling of Swell-Strength of Expansive Soils Using Artificial Intelligence Approaches: ANN, ANFIS and GEP. J. Environ. Manag..

[46] Iqbal M., Zhang D., Jalal F.E., Faisal Javed M. (2021). Computational AI Prediction Models for Residual Tensile Strength of GFRP Bars Aged in the Alkaline Concrete Environment. Ocean Eng..

[47] Murad Y., Tarawneh A., Arar F., Al-Zu’bi A., Al-Ghwairi A., Al-Jaafreh A., Tarawneh M. (2021). Flexural Strength Prediction for Concrete Beams Reinforced with FRP Bars Using Gene Expression Programming. Structures.

[48] Kaloop M.R., Samui P., Iqbal M., Hu J.W. (2022). Soft Computing Approaches towards Tensile Strength Estimation of GFRP Rebars Subjected to Alkaline-Concrete Environment. Case Stud. Constr. Mater..

[49] Ferreira C. (2001). Gene Expression Programming: A New Adaptive Algorithm for Solving Problems. arXiv.

[50] Cevik A. (2007). A New Formulation for Longitudinally Stiffened Webs Subjected to Patch Loading. J. Constr. Steel Res..

[51] Kayadelen C. (2011). Soil Liquefaction Modeling by Genetic Expression Programming and Neuro-Fuzzy. Expert Syst. Appl..

[52] Teodorescu L., Sherwood D. (2008). High Energy Physics Event Selection with Gene Expression Programming. Comput. Phys. Commun..

[53] Raja M.N.A., Shukla S.K., Khan M.U.A. (2021). An Intelligent Approach for Predicting the Strength of Geosynthetic-Reinforced Subgrade Soil. Int. J. Pavement Eng..

[54] Khan M.U.A., Shukla S.K., Raja M.N.A. (2022). Load-Settlement Response of a Footing over Buried Conduit in a Sloping Terrain: A Numerical Experiment-Based Artificial Intelligent Approach. Soft Comput..

[55] Khan M.U.A., Shukla S.K., Raja M.N.A. (2021). Soil–Conduit Interaction: An Artificial Intelligence Application for Reinforced Concrete and Corrugated Steel Conduits. Neural Comput. Appl..

[56] Bardhan A., Kardani N., Alzo’ubi A.K., Samui P., Gandomi A.H., Gokceoglu C. (2022). A Comparative Analysis of Hybrid Computational Models Constructed with Swarm Intelligence Algorithms for Estimating Soil Compression Index. Arch. Comput. Methods Eng..

[57] Topal U., Goodarzimehr V., Bardhan A., Vo-Duy T., Shojaee S. (2022). Maximization of the Fundamental Frequency of the FG-CNTRC Quadrilateral Plates Using a New Hybrid PSOG Algorithm. Compos. Struct..

[58] Kingston G.B., Maier H.R., Lambert M.F. (2005). Calibration and Validation of Neural Networks to Ensure Physically Plausible Hydrological Modeling. J. Hydrol..

[59] Aamir M., Tolouei-Rad M., Vafadar A., Raja M.N.A., Giasin K. (2020). Performance Analysis of Multi-Spindle Drilling of Al2024 with TiN and TiCN Coated Drills Using Experimental and Artificial Neural Networks Technique. Appl. Sci..

[60] Azim I., Yang J., Javed M.F., Iqbal M.F., Mahmood Z., Wang F., Liu Q. (2020). feng Prediction Model for Compressive Arch Action Capacity of RC Frame Structures under Column Removal Scenario Using Gene Expression Programming. Structures.

[61] Shah M.I., Javed M.F., Abunama T. (2021). Proposed Formulation of Surface Water Quality and Modelling Using Gene Expression, Machine Learning, and Regression Techniques. Environ. Sci. Pollut. Res..

[62] Raja M.N.A., Shukla S.K. (2021). Predicting the Settlement of Geosynthetic-Reinforced Soil Foundations Using Evolutionary Artificial Intelligence Technique. Geotext. Geomembr..

[63] Iqbal M.F., Liu Q.F., Azim I., Zhu X., Yang J., Javed M.F., Rauf M. (2020). Prediction of Mechanical Properties of Green Concrete Incorporating Waste Foundry Sand Based on Gene Expression Programming. J. Hazard. Mater..

